# iTRAQ-Based Proteomic Analysis of Dentate Gyrus in Temporal Lobe Epilepsy With Hippocampal Sclerosis

**DOI:** 10.3389/fneur.2020.626013

**Published:** 2021-01-25

**Authors:** Wenbiao Xiao, Zhiquan Yang, Xiaoxin Yan, Li Feng, Lili Long, Tian Tu, Na Deng, Wenjuan Chen, Bo Xiao, Hongyu Long, Yi Zeng

**Affiliations:** ^1^Department of Neurology, Xiangya Hospital, Central South University, Changsha, China; ^2^Department of Neurosurgery, Xiangya Hospital, Central South University, Changsha, China; ^3^Department of Anatomy and Neurobiology, Xiangya School of Medicine, Central South University, Changsha, China; ^4^Department of Psychiatry, Sir Run Run Shaw Hospital, Zhejiang University School of Medicine, Hangzhou, China; ^5^Department of Geriatrics, Second Xiangya Hospital, Central South University, Changsha, China

**Keywords:** temporal lobe epilepsy, hippocampal sclerosis, dentate gyrus, quantitative proteomics, iTRAQ

## Abstract

Temporal lobe epilepsy (TLE) is the most frequent type of focal epilepsy in adults, typically resistant to pharmacological treatment, and mostly presents with cognitive impairment and psychiatric comorbidities. The most common neuropathological hallmark in TLE patients is hippocampal sclerosis (HS). However, the underlying molecular mechanisms involved remain poorly characterized. The dentate gyrus (DG), one specific hippocampal subarea, structural and functional changes imply a key involvement of the DG in the development of TLE. In this study, a isobaric tags for relative and absolute quantitation (iTRAQ)-based quantitative proteomic technique was performed for the analysis of hippocampal DG obtained from patients with TLE-HS compared to control samples obtained from autopsy. Our proteomic data identified 5,583 proteins, of which 82 proteins were upregulated and 90 proteins were downregulated. Bioinformatics analysis indicated that differentially expressed proteins were enriched in “synaptic vesicle,” “mitochondrion,” “cell-cell adhesion,” “regulation of synaptic plasticity,” “ATP binding,” and “oxidative phosphorylation.” Protein-protein interaction network analysis found a pivotal module of 10 proteins that were related to “oxidative phosphorylation.” This study has investigated proteomic alterations in the DG region of TLE-HS patients, and paved the way for the better understanding of epileptogenesis mechanisms and future therapeutic intervention.

## Introduction

Temporal lobe epilepsy (TLE) is the most frequent type of focal (partial) epilepsy in adults (1). The most common neuropathological hallmark in TLE patients is hippocampal sclerosis (HS), and seizures of TLE patients with HS (TLE-HS) are typically resistant to pharmacological treatment (2). Moreover, TLE-HS patients mostly present with cognitive impairment and psychiatric comorbidities (3), mandating the research for the better understanding of underlying epileptogenesis and progression mechanisms. Initial precipitating injury events, such as febrile convulsions, inflammation, or trauma may be proposed as a driving force in the development of HS-associated TLE (4, 5). HS is characterized pathologically by the segmental loss of pyramidal neurons, granule cell dispersion (GCD), and reactive gliosis in the hippocampus (2). The structural and functional changes of the dentate gyrus (DG), one specific hippocampal subarea, including GCD, mossy fiber sprouting, aberrant neurogenesis, and increased excitation, imply a key involvement of the DG in the development of TLE (6, 7).

Although the structural alterations in TLE-HS have been widely documented, the underlying molecular mechanisms involved, however, remain poorly characterized. Proteomics has been an important methodology for studying the changes of the protein profiles in physiological and pathological conditions, providing comprehensive knowledge about molecular patterns and functional pathway alterations. To date, several proteomic studies have examined the protein changes associated with TLE in the hippocampus, as opposed to specific subregions of the hippocampus. Identified differentially expressed proteins (DEPs) were related to mitochondria and bioenergetics, neuronal excitability, and cytoskeleton (8–10). Given the complex and heterogeneous architecture of the hippocampus, studying whole hippocampal specimens may hinder the detection of the distinct changes seen in DG that can only be explained by molecular changes occurring within DG. Additionally, to our knowledge, few previous studies have performed quantitative proteomics in the DG of TLE-HS patients. Thus, we aim to investigate the molecular pathogenesis mechanisms that may occur in the DG of TLE-HS.

Isobaric tags for relative and absolute quantitation (iTRAQ) is a robust quantitative proteomic technique that can simultaneously analyze up to 8-plex samples, and have been widely applied in clinical research (11). In the present study, iTRAQ-based tandem mass spectrometry was performed to undertake proteomic analysis of hippocampal DG obtained from patients with TLE-HS compared to the control samples obtained from autopsy. Our proteomic approach may shed light on the molecular changes associated with HS in DG, that could be used for a better understanding of the specific pathomechanisms of HS and identifying potential therapeutic targets.

## Materials and Methods

### Patients and Tissue Preparation

The study was carried out on seven TLE patients subjected to standard hippocampectomy at the Department of Neurosurgery of Xiangya Hospital. All patients provided their comprehensive medical history, and went through a physical examination, brain magnetic resonance imaging (MRI), and electroencephalograph (EEG). The inclusion criteria of TLE were according to our previous research (12). Preoperative MRI and routine postoperative histopathologic examination of resected tissue confirmed HS. Written informed consent was obtained from all enrolled participants. The study was conducted following the guideline for research involving humans, and approved by the Ethics Committee of Central South University, Xiangya School of Medicine and the affiliated Xiangya Hospital.

Hippocampal samples of the control group were obtained from autopsied patients of Xiangya School of Medicine, and the exclusion criteria were patients with neuropsychiatric disorders and abnormities in the central nervous system (CNS) based on clinical history or histological examination.

**Figure 1:**
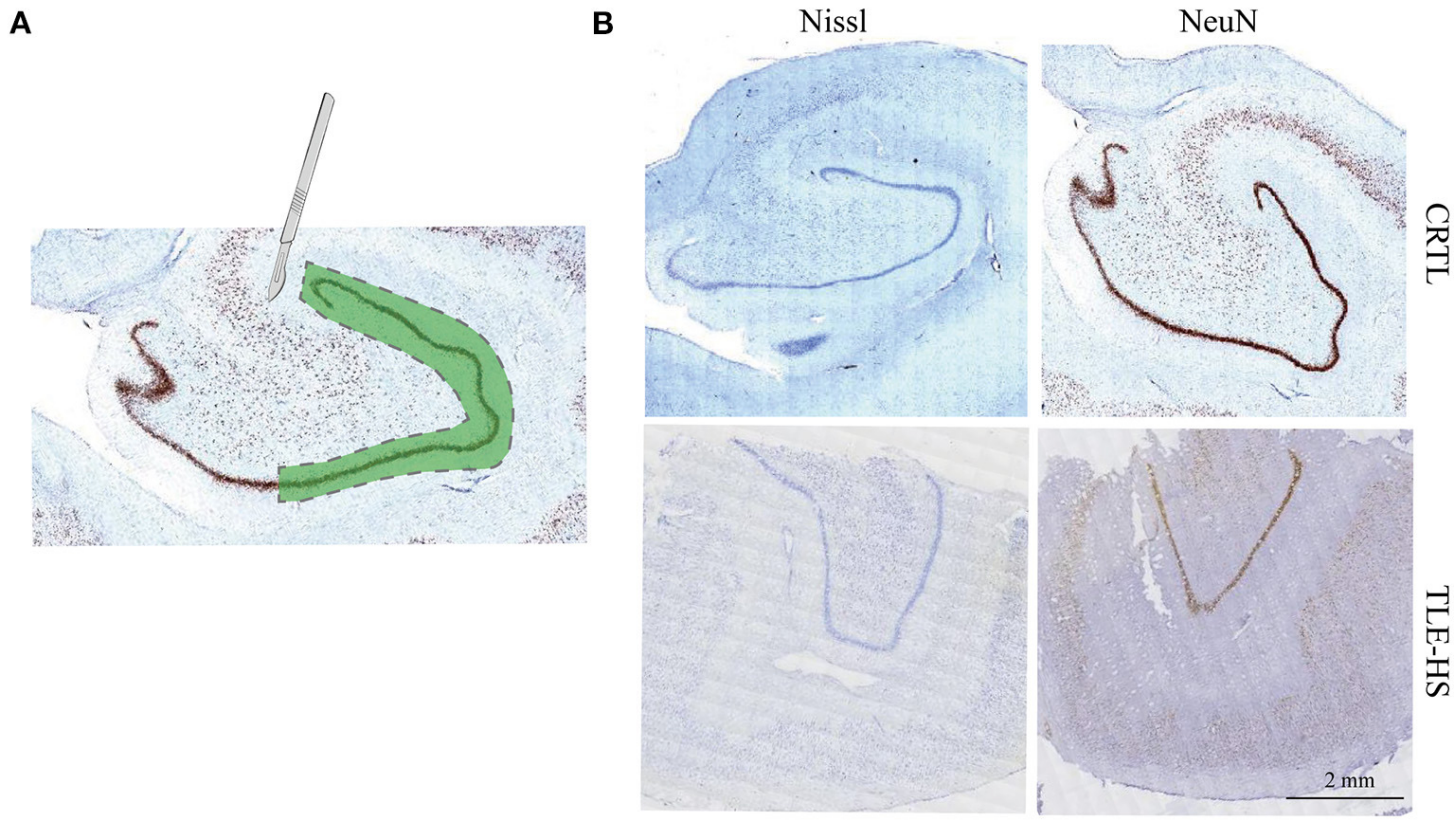
Isolation of dentate gyrus and immunohistochemical staining. **(A)**. Schematic drawing depicting the dissected dentate gyrus region that was used for the proteomics study from the human hippocampus. The dashed lines indicate the cut area. **(B)**. Immunohistochemical staining against NeuN and Nissl staining indicate marked neuron loss of dentate gyrus. Calibration bar = 2 mm.

The resected brain tissues were snap-frozen in liquid nitrogen and then stored at −80°C. Immunohistochemical staining against NeuN (Merck-Millipore, MAB377, 1:1,000) was performed in the brain slices as described before (13), and for Nissl staining, brain slices were placed under agitation in a cresyl violet staining solution. The staining results were used as localization references, an experienced pathologist therefore separated the DG from the rest of the hippocampus by microdissection (Figure 1).

### iTRAQ-8plex Labeling and Mass Spectrometry

Quantitative 8-plex iTRAQ proteomic analysis was carried out on eight independent protein samples. Total proteins were extracted with iTRAQ lysis buffer, processed by disulfide bond cleavage and reductive alkylation of proteins with DTT and iodoacetamide, and then the proteins were hydrolyzed by trypsin. The pooled samples were labeled using the iTRAQ kit according to the manufacturer's protocol (Applied Biosystems Sciex).

The iTRAQ-labeled samples were mixed and resuspended in buffer A (2% acetonitrile, pH 10) and eluted with a 2–95% linear gradient of buffer B (90% acetonitrile, pH 10) over 90 min with a flow rate of 1 mL/min.

The samples were separated into 20 fractions on a C18 column with the HPLC system (Thermo Fisher Scientific) and analyzed using a Q Exactive Plus hybrid quadrupole-orbitrap Mass Spectrometer (Thermo Fisher Scientific). The mass spectrometry proteomics data are available via ProteomeXchange with identifier PXD023048.

### Proteomic Data Analysis and Differentially Expressed Proteins Identification

Tandem mass spectra were searched for computational analysis in the Homo sapiens protein database using the Proteome Discoverer software. The Sequest algorithm was used to search with a fragment ion mass tolerance of 0.02 Da and a parent ion tolerance of 15 PPM. An FDR of <1% and with at least two unique peptides were used in identifying and quantifying proteins. The analysis of covariance (ANCOVA) model was hired for further analysis adjusting for age. The protein threshold was set to achieve 95% confidence, a *P*-value and fold-change cut-offs were used to classify an upregulated protein (*p* < 0.01 and fold-change >2) or a downregulated protein (*p* < 0.01 and fold-change <0.5). A volcano plot and hierarchical cluster analysis were performed with the software R (Version: 3.5.1).

### Bioinformatic Analysis

DAVID Bioinformatics Resources 6.8 (https://david.ncifcrf.gov/) was used to conduct enrichment analysis of DEPs and module proteins (14, 15), and the underlying mechanism was identified in the context of Kyoto Encyclopedia of Genes and Genomes (KEGG) pathways and gene ontology (GO) terms, which consisted of cellular components (CC), molecular functions (MF), and biological processes (BP). *p*-value <0.05 and gene count ≥2.0 were set as the threshold of significance.

A protein-protein interaction (PPI) network was constructed using STRING (https://string-db.org/). Based on the STRING database, the interaction network was then visualized by Cytoscape software 3.0. Moreover, the Molecular Complex Detection (MCODE) app in Cytoscape was utilized to screen the entire PPI network for hub genes modules, with a degree cutoff ≥2, node score cut-off ≥0.2, K-Core ≥2, and Max Depth = 100.

## Results

### Demographics and Clinical Characters of Individuals

A total of 14 participants (including seven patients with TLE and seven autopsy controls) were recruited to our study. As is shown in Table 1, the age of the patients with TLE ranged from 9 to 42 years old (25.2 ± 9.2 year) with two female cases and five male cases. The duration of having seizures in the TLE patients was 8.8 ± 5.4 years. Of the sampled autopsy tissue (2 female, 5 male), the age of the subjects at death ranged from 14 to 77 years (50.5 ± 19.9 year). The demographic and clinical data of controls are also shown in Table 1.

**Table 1:** Clinical and demographic data of patients with MTLE and autopsied subjects (control samples).

**Data**	**MTLE patients**	**Autopsied subjects**
Number of samples	7	7
Gender-female	2	2
Age at surgery or autopsy-year (mean ± SD)	25.2 ± 9.2	50.5 ± 19.9
Seizure duration-year (mean ± SD)	8.8 ± 5.4	N/A
Presence of febrile seizure	1	None
Family history of epilepsy	1	None
Postmortem period	n/a	<10 h
Changes in central nervous system	Hippocampal sclerosis	None

### Differentially Expressed Proteins Revealed by iTRAQ Analysis

**Figure 2:**
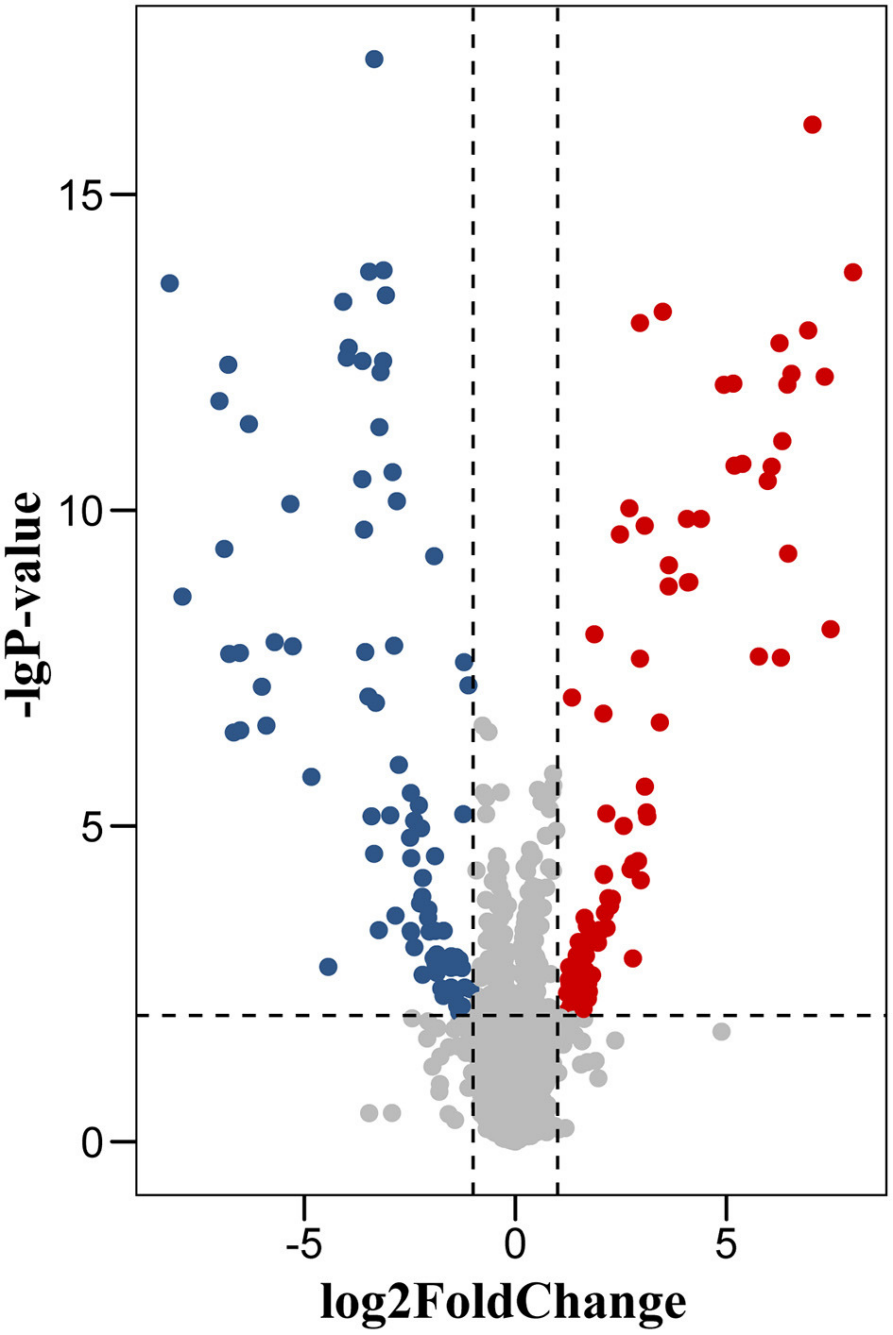
Volcano plot of protein changes in dentate gyrus between TLE-HS patients and control groups. There were 172 proteins with a fold-change of >2 or <0.5 and *p*-values <0.01 were identified as the significant difference. Red dots represent upregulated proteins, blue dots are downregulated proteins.

**Figure 3:**
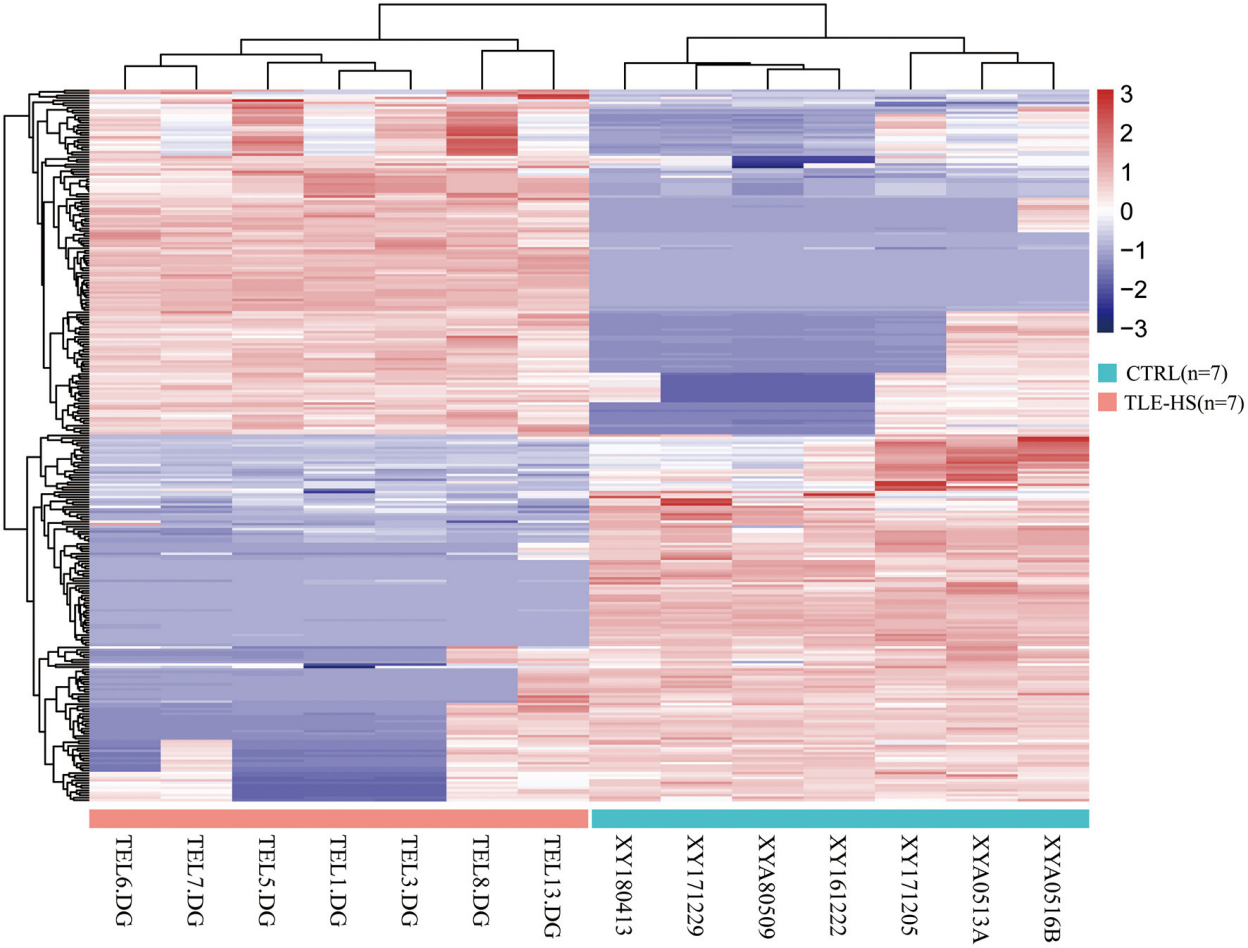
Hierarchical cluster analysis of 172 differentially expressed proteins in TLE-HS and control groups. Each protein expression level from different samples is presented horizontally, and the expression levels of all proteins in each sample are listed vertically. The log2-transformed expression levels are shown in a red-blue color scale (red is upregulated, blue is downregulated). The pink color in the heatmaps indicates TLE-HS patients, the blue color indicates control samples.

We identified a total of 5,583 proteins by the iTRAQ experiments. A total of 5,234 proteins were quantified, among which 172 proteins were differentially expressed (*p* < 0.05, fold-change >2 or <0.5), including 82 upregulated and 90 downregulated proteins (Figure 2, Supplementary Table 1). The 10 most DEPs (upregulated and downregulated) are listed in Table 2. The visualization of hierarchical cluster analysis results revealed a distinction in DG region proteins between TLE-HS patients and control samples (Figure 3).

**Table 2:** Top 10 upregulated and downregulated proteins in MTLE patients compared to controls by iTRAQ.

**Gene**	**Protein name**	**Fold-change**	***P*-value**	**Changes**
FCHO2	F-BAR domain only protein 2 isoform X7	256.14	2.16E-13	UP
STXBP1	Syntaxin-binding protein 1 isoform b	177.09	3.08E-08	UP
TATDN1	Putative deoxyribonuclease TATDN1 isoform d	160.63	4.23E-12	UP
MPZL1	Myelin protein zero-like protein 1 isoform b precursor	131.58	1.53E-15	UP
NDUFA5	NADH dehydrogenase [ubiquinone] 1 alpha subcomplex subunit 5 isoform 1	122.48	1.08E-12	UP
SVOP	Synaptic vesicle 2-related protein	93.36	4.27E-12	UP
ATP5ME	ATP synthase subunit e, mitochondrial	88.21	2.01E-09	UP
NUP50	Nuclear pore complex protein Nup50 isoform a	87.22	8.44E-12	UP
MAP4K2	Mitogen-activated protein kinase kinase kinase kinase 2 isoform X7	80.07	5.16E-11	UP
RIN1	Ras and Rab interactor 1 isoform 3	78.40	4.75E-08	UP
RBBP4	Histone-binding protein RBBP4 isoform a	0.003	1.83E-13	DOWN
UQCR10	Cytochrome b-c1 complex subunit 9 isoform a	0.004	1.1E-08	DOWN
EGLN1	Egl nine homolog 1	0.008	1.52E-11	DOWN
GSTT2	Glutathione S-transferase theta-2 isoform b	0.008	1.92E-09	DOWN
SNAPIN	SNARE-associated protein Snapin	0.009	3.27E-12	DOWN
NDUFS5	NADH dehydrogenase [ubiquinone] iron-sulfur protein 5	0.009	6.33E-08	DOWN
LRRTM1	Leucine-rich repeat transmembrane neuronal protein 1 precursor	0.010	1.05E-06	DOWN
RPS17	40S ribosomal protein S17	0.011	6.58E-08	DOWN
MTFR1L	Mitochondrial fission regulator 1-like isoform a	0.011	1.38E-08	DOWN
ABCF2	ATP-binding cassette sub-family F member 2 isoform a	0.013	3.05E-11	DOWN

### Gene Ontology, KEGG Pathway Analysis of Differentially Expressed Proteins

**Figure 4:**
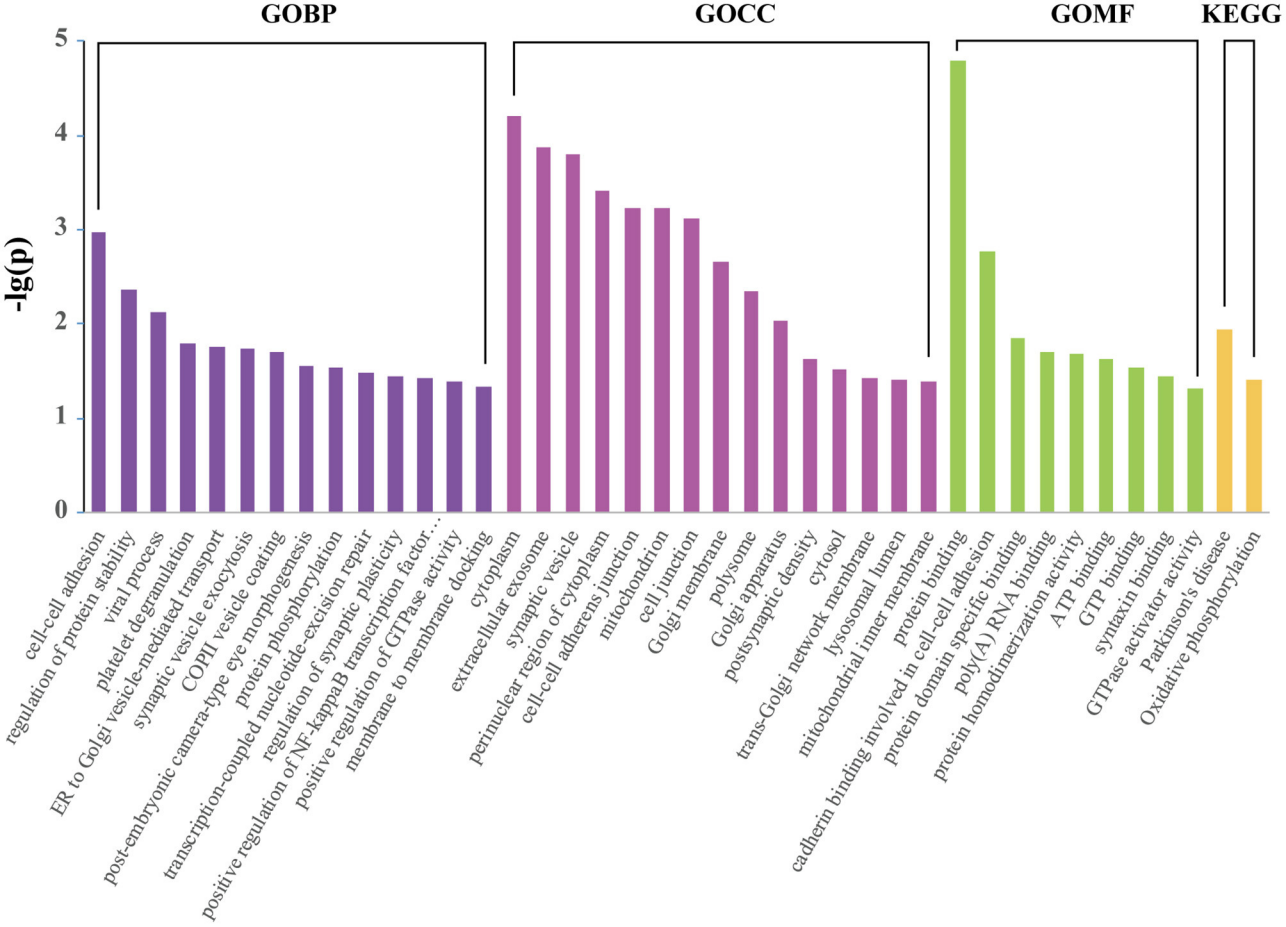
Enriched KEGG pathway and GO analysis of 172 differentially expressed proteins using DAVID. The identified proteins were annotated with DAVID into molecular function, biological process, cellular component, and KEGG pathway.

GO terms were further assigned to DEPs according to their CC, MF, and BP (Figure 4). From the perspective of CC, it can be noticed that the top DEPs were enriched in “cytoplasm,” “extracellular exosome,” “synaptic vesicle,” and “mitochondrion.” From the BP perspective, the most enriched terms included “cell-cell adhesion,” “regulation of protein stability,” “synaptic vesicle exocytosis,” and “regulation of synaptic plasticity.” As for MF, “protein binding,” “ATP binding,” and “syntaxin binding” were the most enriched terms. “Parkinson's disease” and “oxidative phosphorylation” were the two significantly enriched KEGG pathways (Figure 4). Functional enrichment analysis of up and downregulated proteins are presented in Supplementary Table 2.

### PPI Network Construction and Analysis of Modules

**Figure 5:**
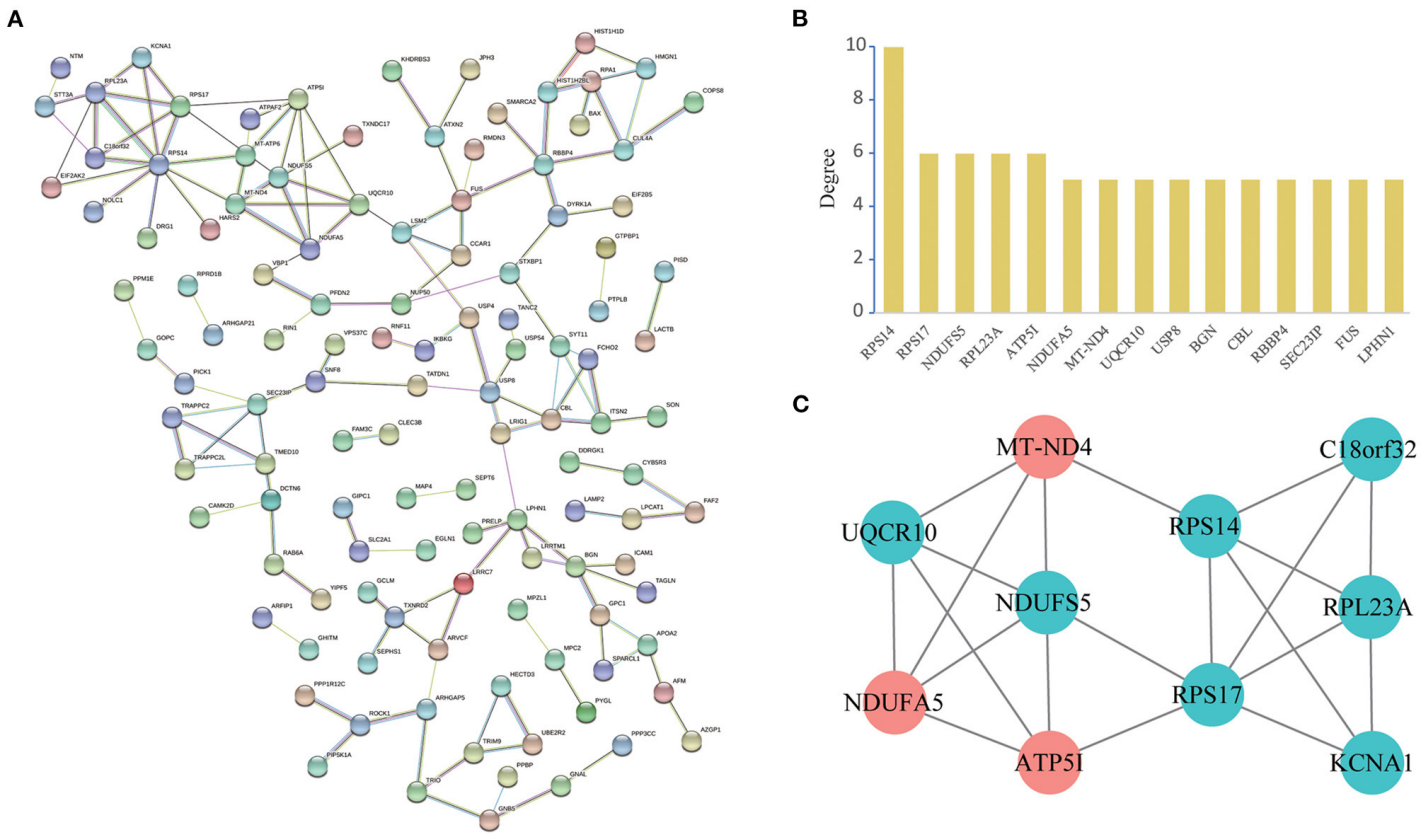
PPI network and module analysis. **(A)**: Protein-protein interaction network of differentially expressed genes by the STRING database. We identified 144 PPI pairs among 119 differentially expressed genes. **(B)**: Degree distribution histogram of hub genes. **(C)**: A significant module selected from the entire PPI network using the Cytoscape MCODE app with the default parameters (Degree cutoff ≥2, Node score cut-off ≥0.2, K-Core ≥2 and Max Depth = 100). Red nodes represent upregulated proteins and blue nodes represent downregulated proteins.

A total of 119 nodes and 144 edges were selected to plot the PPI network by STRING, which consisted of 82 upregulated proteins and 90 downregulated proteins (Figure 5A). We counted up the degree of the protein to highlight specific proteins with top linkage (Figure 5B).

Subsequently, in the entire PPI network, a pivotal module of 10 proteins (RPS14, RPS17, NDUFS5, RPL23A, ATP5I, NDUFA5, MT-ND4, UQCR10, KCNA1, C18orf32) was identified with the default parameters using MCODE (Figure 5C). Moreover, it is worth noting that, functional and KEGG pathway enrichment analysis revealed that genes in this module were found to relate to “oxidative phosphorylation,” “Parkinson's disease,” “metabolic pathways,” and “mitochondrial respiratory chain complex” (Table 3).

**Table 3:** Functional enrichment analysis of differentially expressed genes in the module.

**Category**	**Term**	***P*-value**	**Genes**
KEGG_PATHWAY	Oxidative phosphorylation	4.47E-06	NDUFA5, UQCR10, NDUFS5, ND4, ATP5I
KEGG_PATHWAY	Parkinson's disease	2.84E-04	NDUFA5, UQCR10, NDUFS5, ND4
KEGG_PATHWAY	Ribosome	0.007635	RPS17, RPS14, RPL23A
KEGG_PATHWAY	Non-alcoholic fatty liver disease (NAFLD)	0.00935	NDUFA5, UQCR10, NDUFS5
KEGG_PATHWAY	Alzheimer's disease	0.011486	NDUFA5, UQCR10, NDUFS5
KEGG_PATHWAY	Huntington's disease	0.014838	NDUFA5, UQCR10, NDUFS5
KEGG_PATHWAY	Metabolic pathways	0.021831	NDUFA5, UQCR10, NDUFS5, ND4, ATP5I
GOTERM_BP	Mitochondrial electron transport, NADH to ubiquinone	2.96E-04	NDUFA5, NDUFS5, ND4
GOTERM_BP	Mitochondrial respiratory chain complex I assembly	4.90E-04	NDUFA5, NDUFS5, ND4
GOTERM_BP	SRP-dependent co-translational protein targeting to membrane	0.001088	RPS17, RPS14, RPL23A
GOTERM_BP	Viral transcription	0.001539	RPS17, RPS14, RPL23A
GOTERM_BP	Nuclear-transcribed mRNA catabolic process, nonsense-mediated decay	0.001735	RPS17, RPS14, RPL23A
GOTERM_BP	Translational initiation	0.002291	RPS17, RPS14, RPL23A
GOTERM_BP	rRNA processing	0.005487	RPS17, RPS14, RPL23A
GOTERM_BP	Translation	0.007591	RPS17, RPS14, RPL23A
GOTERM_BP	Ribosomal small subunit assembly	0.01014	RPS17, RPS14
GOTERM_CC	Mitochondrial inner membrane	2.19E-05	NDUFA5, UQCR10, NDUFS5, ND4, ATP5I
GOTERM_CC	Mitochondrial respiratory chain complex I	1.96E-04	NDUFA5, NDUFS5, ND4
GOTERM_CC	Ribosome	0.002228	RPS17, RPS14, RPL23A
GOTERM_CC	Mitochondrion	0.016474	NDUFS5, ND4, RPS14, ATP5I
GOTERM_CC	Cytosolic small ribosomal subunit	0.020882	RPS17, RPS14
GOTERM_MF	NADH dehydrogenase (ubiquinone) activity	2.81E-04	NDUFA5, NDUFS5, ND4
GOTERM_MF	Structural constituent of ribosome	0.005832	RPS17, RPS14, RPL23A

## Discussion

For patients with refractory epilepsy, surgical therapy is an important alternative treatment, and surgical resection of the epileptogenic zone offers valuable materials for investigating the molecular mechanisms underlying epilepsy. Studies in surgical specimens revealed the critical role of the hippocampus for TLE and, particularly, of the DG in several pathological processes that were related to the epileptogenesis and development of the disease (16). However, the proteomics alterations of DG in TLE-HS patients have not been well-studied. In this regard, we utilized iTRAQ-based tandem mass spectrometry to resolve and compare the protein expression of DG to characterize the molecular changes associated with HS pathology. Differential expression analysis identified 82 upregulated proteins and 90 downregulated proteins in the samples with TLE-HS compared to the autopsy samples. Subsequent functional analysis and PPI network analysis suggested that these proteins might be involved in biological changes that relate to epilepsy.

Our results showed that oxidative phosphorylation and metabolic pathways were significantly enriched KEGG pathways. The proteins that participate in those two pathways included NDUFA5, UQCR10, NDUFS5, ND4, and ATP5I. Among that, NDUFA5, UQCR10, NDUFS5, and ATP5I were also in the top 10 dysregulated proteins list, and NDUFA5, UQCR10, NDUFS5, ND4, and ATP5I were components of the significant module in the PPI network. ATP5I and NDUFA5 have been reported to be dysregulated in the HS of TLE patients and animal models (17–19). Oxidative phosphorylation takes place in the mitochondria of eukaryotic cells, and couples the process with the oxidation of substrates and ATP generation (20). Since mitochondrial oxidative phosphorylation meets the energy demands in neurons and mitochondria participate in cellular Ca^2+^ homeostasis, mitochondria malfunction has a strong impact on synaptic transmission and neuronal excitability, which has been proposed to play a pivotal role in epileptogenesis (21). Moreover, human and experimental TLE with a variety of impaired mitochondrial functions have been reported (22). Additionally, mitochondrial dysfunction is highly relevant for neuronal loss, which is a distinct pathological feature in TLE-HS patients (23).

There was also significant enrichment of several KEGG pathways associated with neurodegeneration: Parkinson's disease, Alzheimer's disease, and Huntington's disease. The overlap proteins of these pathways included NDUFA5, NDUFS5, ND4, which are subunits of complex I (CI; NADH-ubiquinone oxidoreductase) (24). CI is the first complex of the mitochondrial oxidative phosphorylation system, deficiencies in which have been associated with TLE, Huntington's disease, and Parkinson's disease (25–27). Humans and animals exposed to the CI inhibitor develop Parkinsonism, which strengthened CI deficiencies in Parkinson's disease (28, 29). Ketone bodies metabolism actively produces energy in periods of energy shortage in the brain, which may also be part of the compensatory mechanism of CI deficiencies (30). Ketogenic diet has been shown to be involved in energy metabolism, and to stimulate mitochondrial biogenesis (31). Moreover, ketogenic diet induced a coordinated upregulation of mitochondrial genes, exhibited neuroprotective effects in the hippocampus of a TLE model (32, 33), and reduced the amyloid pathology in an experimental model of Alzheimer's disease (34). Although these neurodegenerative diseases differ in their underlying etiology, they share a common feature, in that each disorder implicates a certain mitochondrial dysfunction through a specific pathway. Above all, this perspective may raise the possibility for the development of novel neuroprotective agents in addition to the conventional anticonvulsant therapy of TLE.

While 172 DEPs were identified, we believe that the following selection may be of interest for further investigation. Three proteins related to neurotransmitter release, STXBP1, SNAPIN, and SVOP, also appeared in the top 10 dysregulated proteins list. Synaptic transmission is critical for normal brain functioning and homeostasis, and abnormal regulation could break the delicate balance between excitatory and inhibitory synapses, which causes seizures (35). *STXBP1* encodes a syntaxin1-binding protein, and helps regulate the release of neurotransmitters through the regulation of transmembrane attachment protein receptors (syntaxin1) (36). *STXBP1* gene mutations have been reported to cause STXBP1 encephalopathy or early infantile epileptic encephalopathy-4 (EIEE4) (37). The synaptic vesicle glycoprotein 2 (SV2) family and SV2-related proteins (SVOP) are located in synaptic neurotransmitter-containing vesicles, and regulate the secretion of neurotransmitters from presynaptic neurons, possibly via synaptic vesicle recycling (38, 39). SV2A is the target site of levetiracetam (LEV) and related compounds, which are approved for use in patients with multiple types of seizures and epilepsies (40, 41). Although SVOP is structurally similar to SV2, little is known about its function (39). Bando et al. noted that the expression of *SVOP* was significantly upregulated in the hippocampus of refractory mesial TLE patients (42). KCNA1, which appeared in the significant module of the PPI network, encoded a voltage-gated potassium channel that mediates primarily potassium transport in excitable membranes of the CNS, and contributes to the regulation of neuronal excitability especially in mossy fibers of the hippocampus (43, 44). KCNA1^−/−^ mice display early seizures and premature unexpected death, and can be used as a valuable sudden unexpected death (SUDEP) model in epilepsy (45). Notably, KCNA1 is the target of Isoflurane, a commonly used inhalation agent, which has anticonvulsant properties and can terminate refractory status epilepticus in patients (46).

Previous proteomic research analyzing samples of patients with epilepsy have identified different differential protein profiles. This is because of (i) different types of epilepsy; (ii) diverse sample sources or brain regions; and (iii) different controls. Two previous studies have performed proteomics on the DG region of epilepsy patients: Liu et al. (47) compared the proteomes of basal and dispersed granule cells in the hippocampus of MTLE patients with GCD (not including surgical or post-mortem healthy controls), and found that upregulated proteins in dispersed samples were involved in developmental cellular migratory processes. Pire et al. (48) performed proteomic analysis of the hippocampal CA1-3 region, frontal cortex, and DG samples from epilepsy (without specific classification) and control cases, the pathway analysis of altered proteins in the DG involved in mitochondrial dysfunction showed the most enrichment. Interestingly, by analyzing the gene expression profiles of dentate granule cells from surgically resected hippocampal specimens from patients with MTLE with and without HS, Griffin et al. (19) demonstrated that differentially expressed genes were associated with the oxidative phosphorylation pathway, which is generally consistent with our findings. Our proteomic study further expanded the biomedical resource for molecular mechanism research on epilepsy. Together, more detailed research including different brain subregions, specific cell components, and even single-cell sequencing would offer more promising options to bridge the understanding of epileptogenesis from a different perspective.

However, several points should be taken into account when analyzing and interpreting the results. Firstly, subjects enrolled in our study appear to be refractory patients as they still have seizures even after combined antiepileptic drugs (AEDs) treatment; AEDs may have an impact on the proteome of the brain tissue, however, no published literature has explored this. Second, the age of patients with TLE was younger than that of control subjects, which may still be a tentatively confounding factor after an adjustment. Third, it is not possible to use samples with identical post-mortem times for ethical and practical reasons, but we have to collect samples from autopsies within a maximum post-mortem time of 10 h. Fourth, tissue samples from distinct sample acquisition methodologies for TLE and control groups (hippocampectomy and autopsy) may induce possible effects in protein content integrity. Therefore, control samples with a smaller post-mortem time were selected as much as possible, and protein samples were subjected to strict quality control. Last, as a limited quantity of DG brain tissue was obtained from each subject, proteins profiled by iTRAQ-based tandem mass spectrometry were not verified by immunohistochemistry assays or western blot, which should warrant further validation in larger independent samples.

## Conclusion

For the first time we applied an iTRAQ-based proteomic study to investigate the molecular alterations in the DG region of TLE-HS patients and autopsy controls. Our proteomic work revealed a number of dysregulated proteins and involved critical biological pathways associated with HS pathological mechanisms. The gained information will pave the way to a better understanding of epileptogenesis and future therapeutic intervention.

## Data Availability Statement

The mass spectrometry proteomics data have been deposited to the ProteomeXchange Consortium via the PRIDE partner repository with the dataset identifier PXD023048.

## Ethics Statement

The studies involving human participants were reviewed and approved by the Ethics Committee of Central South University, Xiangya School of Medicine and the affiliated Xiangya Hospital. Written informed consent to participate in this study was provided by the participants' legal guardian/next of kin.

## Author Contributions

WX, HL, and YZ contributed to the study conception and design. WX, ZY, XY, TT, HL, LL, and LF collected the patients' data. WX, HL, ND, and WC analyzed and interpreted the data. WX, YZ, WC, and BX funded the study. All authors contributed to writing the article and approved the submitted version.

## Conflict of Interest

The authors declare that the research was conducted in the absence of any commercial or financial relationships that could be construed as a potential conflict of interest.
